# Message-based psychotherapy for older adults: A cohort comparison study

**DOI:** 10.3389/fpsyt.2022.951354

**Published:** 2022-08-25

**Authors:** Patrick J. Raue, Nicole Fridling, Jiyoung Song, Thomas D. Hull, George S. Alexopoulos, Patricia A. Arean

**Affiliations:** ^1^Department of Psychiatry & Behavioral Sciences, University of Washington Medical Center, Seattle, WA, United States; ^2^Talkspace, New York, NY, United States; ^3^Department of Psychology, University of California, Berkeley, Berkeley, CA, United States; ^4^Department of Psychiatry, Weill Cornell Medical College, White Plains, NY, United States

**Keywords:** older adult, telemedicine, digital mental health interventions, depression, anxiety

## Abstract

**Objective:**

Digital Mental Health Interventions (DMHI) can diminish inequities in mental health care provision. As DMHIs increase in popularity, however, older adults may be unintentionally excluded due to barriers such as lack of awareness, internet access, digital tools, technological socialization and education, physiological accessibility, and communication technology infrastructure. The aim of this study was to examine longitudinal treatment engagement patterns and 15-week clinical outcomes of depressed and anxious older adults compared to a matched cohort of younger adults seeking treatment from a large asynchronous telemedicine provider.

**Methods:**

The 2,470 older adults (55+ years) and a matched cohort of younger adults (26–35 years) diagnosed with depression or anxiety were treated by licensed therapists *via* messaging 5 days a week. Patterns of treatment engagement on the platform were compared across groups by examining total number of days in treatment, days actively messaging on the platform, and average words and messages per week sent by patients over the entire period they remained in treatment. Symptoms were assessed every 3 weeks using the Patient Health Questionnaire (PHQ-9) and the Generalized Anxiety Disorder Scale (GAD-7), and changes were compared across age groups over 15 weeks.

**Results:**

Older patients attended more days in treatment than younger patients, but there were no differences in number of days actively messaging on the platform, number of messages per week, or word count per week. The two age groups did not differ in their final anxiety or depressive symptoms when controlling for total number of weeks attended. Patients in the younger age group experienced a quicker rate of reduction than older adults in their anxiety, but not depressive symptoms.

**Conclusions:**

Among individuals willing to initiate care through a DMHI, older adults had overall similar engagement as younger adults and they showed similar improvement in symptoms of depression and anxiety. Given the advantages of message-based care for aiding a mental health workforce in serving larger numbers of individuals in need and the expected growth of the aging population, these findings could help healthcare systems in evaluating a variety of treatment options and delivery media for meeting the healthcare needs of the future.

## Introduction

Depression and anxiety are leading causes of disability in the US and worldwide, affecting 17% of Americans across age and racial/ethnic groups (1, 2). These conditions are associated with suicide, increased mortality, worsened medical outcomes, cognitive impairment, and economic burden (3–6). While psychotherapies are effective in the treatment of both mid and late-life depression and anxiety (7–9), these conditions remain significant public health concerns.

Difficulties accessing and engaging with psychotherapies significantly reduce their reach and impact in treating depression and anxiety in the general population. Most middle-aged and older adults are treated with antidepressants despite their modest efficacy (10, 11) and noted patient preferences for psychotherapy across age groups (12–15). Difficulty for patients accessing psychotherapy has been attributed to several factors. Traditional delivery of psychotherapy often involves weekly 50-mins visits and can be onerous for people seeking help, especially when they are employed, have caregiver demands, are disabled, or have other medical appointments to manage (12, 14). Moreover, the location of services may be inconvenient. For those who live in rural areas where the closest health center may be hours away (16), weekly hour-long visits are not feasible. Attitudinal barriers such as mental health stigma also interfere with help-seeking from traditional venues (17). Insurance constraints, lack of available mental health professionals, and physical impairments are particularly relevant barriers for older adults (14).

Digital Mental Health Interventions (DMHIs) can address many access barriers. DMHIs involve asynchronous message-based communication *via* text, audio or video message, where patients may message their therapist at any point throughout the week. These interventions have shown early promise for offering evidence-based psychosocial interventions for mental health conditions, with the potential to reach millions of lives (18–23). DMHIs are becoming a widely popular and available form of psychotherapy delivery, with as many as 20 companies now offering this form of care. Although the effectiveness of this method of delivering psychotherapy has not been rigorously evaluated to date, preliminary data suggest that DMHIs are an acceptable approach for younger and mid-life patients and for clinicians who treat common mental health conditions (24–28). Message-based formats also allow for more frequent contact with a psychotherapist when needed throughout the week, which can capitalize on findings that high frequency of contact over time results in faster recovery (29–31).

Despite the promise of DMHIs in diminishing inequities of mental health care provision, however, it is unclear how older adults in particular engage and respond to these formats, and some surveys suggest they may be less amenable to this type of care (32). As DMHIs increase in popularity, older adults may be unintentionally excluded due to several barriers including lack of awareness, internet access, digital tools, technological socialization and education, physiological accessibility, and communication technology infrastructure. Discomfort with technology may lead to stress around communication, generate a perceived inability to use the message-based format correctly, impair trust in the provider and the platform's privacy protections, and reduce the clinical efficacy of the intervention. Investigating how older adults use and respond to DMHIs is particularly timely in the context of two Institute of Medicine reports that predict that the aging population will soon overwhelm the healthcare system (1, 33).

The aim of this study was to conduct a naturalistic longitudinal observation of depressed and anxious older adults (55+) and a matched cohort of younger adults (26–35) seeking treatment from a large asynchronous telemedicine provider. We first compared patterns of treatment engagement across groups by examining treatment retention and average number of words and messages used by patients for the duration of time they chose to engage with the platform. Secondly, we compared reduction in depressive (PHQ-9) and anxiety symptoms (GAD-7) between groups over a 15-week period.

## Materials and methods

### Setting

This study examined the care provided by licensed therapists across the United States through the Talkspace telemedicine platform. Talkspace patients access the site through internet search, Employee Assistance Programs, or individual insurance behavioral health benefits. Intake clinicians first conduct brief, standardized intakes through a live, synchronous messaging session to identify presenting problems, contextual factors, and treatment history, and to assign clinical diagnoses. A matching algorithm presents three clinicians who meet the patient's reported preferences. After the patient chooses among the three options, both participants access a messaging “room” for treatment, and the patient completes a brief self-report baseline assessment including the PHQ-9 and GAD-7.

All study data were collected for organizational quality assurance and program management purposes between January 1, 2016 and July 16, 2021. Patients and therapists gave written consent for use of their data in deidentified, aggregate format as part of the Talkspace user agreement that all parties signed before using the platform. Study data has been classified as exempt by the Institutional Review Board at Teachers College, Columbia University (15–426).

### Participants

Patient participants were those who sought treatment through the Talkspace platform and had completed at least one PHQ-9 or GAD-7 assessment. Inclusion criteria were: English speaking; United States resident; regular internet or smartphone access; diagnosis of unipolar depression or anxiety disorder *via* clinical assessment; and a score >10 on baseline PHQ-9 or GAD-7 scales. Exclusion criteria were: bipolar disorder as determined by clinical assessment; psychosis diagnosis or features; active alcohol or substance abuse; and active suicidal ideation as assessed *via* positive response to any of items 3–6 on the Columbia Suicide Severity Rating Scale Lifetime-Recent Screen. For this study, older adults were defined as 55 years or older (*n* = 2,470). This cutoff has been used in some other studies of late life depression, and the younger spectrum of older adults may be most likely to use DMHIs. Given that individuals at the lower level of this cutoff were within working age range and likely comfortable with computers and smart phone technology, however, we examined the association of age as a continuous variable with key metrics of treatment engagement. To compare the entire sample of older users' platform activity, engagement in treatment, and clinical response, we created a matched sample of randomly selected 25–36 years old patients (*n* = 2,663) who used the platform during the same time-period. See Table 1 for demographic and clinical characteristics.

**Table 1:** Demographic characteristics and treatment engagement (*N* = 4,841).

	**Older sample (*****N*** = **2,470)**			**Matched sample (*****N*** = **2,663)**			**Fisher's exact test**	
**Variable**	***n***	**Valid %**	**Mean (SD)**	***n***	**Valid %**	**Mean (SD)**	**U**	***p*-value**
Age			58.65 (5.30)			29.03 (3.07)		
Gender								
Female	998	75.72%		1,100	75.86%			
Male	315	23.90%		330	22.76%			
Transgender or non-binary	5	0.38%		20	1.38%			
No response	1,152			1,213				
Race/Ethnicity								
African American	10	7.63%		10	11.76%			
Asian	3	2.29%		8	9.41%			
Caucasian	107	81.68%		56	65.88%			0.008583
Hispanic	3	2.29%		4	4.71%			
Native American	1	0.76%		0	0.00%			
Other	4	3.05%		7	8.24%			
Declined	3	2.29%		0	0.00%			
No response	2,339			2,578				
Engagement								
Weekly messages			3.67, Min 1, Max 841 (5.87)			4.87, Min 1, Max 840 (7.41)		
Weekly word count			314.5, Min 1, Max 3,086 (720.2)			336.17, Min 1, Max 4,032 (747.1)		
Time (in days) on platform			93.17, (121.06)			81.06, (96.33)		
Days actively messaging on platform			18.93, Min 1, Max 332 (29.3)			18.43, Min 1, Max 272 (25.1)		

Therapist participants were Master's level or higher, employed by Talkspace, licensed in at least in one state, and experienced delivering mental health care for at least three years post-licensure. There was a total of 2,042 therapists, 84% of whom were female. The majority of therapists identified as Licensed Clinical or Psychiatric Social Workers (29.87%) or Licensed Marriage & Family Therapists (36.29%), and 50% had more than 10 years of experience. According to their self-report, therapists offered treatment based on multiple orientations: 61.0% cognitive-behavioral therapy, 40.3% third-wave cognitive-behavioral interventions (e.g., mindfulness-based therapy), and 25.5% psychodynamic or relational psychotherapy. Therapists were matched only to patients within the state in which they were licensed.

### Intervention

Over a secure, HIPAA-compliant platform accessible on mobile and desktop devices, therapists and patients asynchronously exchanged text, audio, and video-based messages depending on patient preference. Patients were able to send unlimited messages 24/7 for therapists to review during standard working hours. Therapists responded to messages from their patients at least once a day, 5 days a week. Therapists were held to all ethical and professional reporting standards for their respective fields. Referrals were provided for patients deemed to require a higher level of care.

The Talkspace platform automatically counted the number of words and messages sent by patients as metadata. This data was used as a proxy to quantify the level of therapeutic engagement in the asynchronous messaging medium.

### Assessments

Patients were assessed for depression and anxiety symptoms at baseline and then every 3 weeks until they ended treatment. Assessments were introduced to patients as an important part of care to both facilitate goal setting and track progress, but they were not mandatory to participate in treatment. This study analyzed six assessment points: baseline, Week 3, Week 6, Week 9, Week 12, and Week 15. While many psychotherapy RCTs assess outcomes at 12 weeks, we chose 15 weeks as the final assessment point given existence of a reasonable number of completed patient surveys at this time.

Severity of depression was assessed with the 9-item Patient Health Questionnaire (PHQ-9) (34). All item responses were provided in the form of a 4-point Likert scale (0 = *Not at all* to 3 = *Nearly every day*), yielding a total maximum score of 27. With high documented sensitivity and specificity, the threshold for clinical (or moderate) depression is a total score greater or equal to 10.

The 7-item Generalized Anxiety Disorder Scale (GAD-7) was used to assess severity of anxiety symptoms. The scale has a total maximum score of 21; Responses were provided on a 4-point Likert scale (0 = *Not at all* to 3 = *Nearly every day*), yielding a total maximum score of 21. A total score of 10 or above indicates anxiety of at least moderate levels.

When leaving the platform, patients were prompted for their primary reason for discontinuing treatment from a list of options. Reasons included meeting their goals, cost concerns, not liking the therapy medium, a disconnect with their therapist, deciding to pursue traditional face-to-face psychotherapy, and technical issues.

### Analytic plan

To analyze our first aim on patterns of treatment engagement on the platform, we chose total number of days in treatment as the primary outcome. We also tabulated number of days actively messaging on the platform, average words, and average messages per week sent by patients over the entire period they remained in treatment. These variables were later included in the multilevel models presented below. Differences in demographic variables were assessed using Fisher's Exact Test. Following convention, only the p-value for this test was reported. We examined simple correlations between age as a continuous variable and engagement metrics within the older adult sample, to determine whether likely greater comfort with technology among the young-old affected treatment engagement with the DMHI.

To analyze our second aim on clinical outcomes, we chose reduction in symptom severity as the primary outcome over the course of 15 weeks (PHQ-9 or GAD-7 scores, depending on which assessment scores were elevated >10 at baseline). Patients needed to have at least one follow-up assessment to be included in these analyses. Symptom reduction was monitored separately for depression and anxiety. The depression model included patients with elevated PHQ-9 scores with or without elevated GAD-7 scores, and the anxiety model patients with elevated GAD-7 scores with or without elevated PHQ-9 scores. Thus, those who had elevated scores on both scales were included in both models (66.1% of older adults and 71.5% of younger adults).

To compare symptom reduction between the two age cohorts, we used the *nlme* (36) package to construct multilevel linear regression models. Multilevel modeling was suitable for the nested structure of our dataset (repeated measures within patients). According to the unconditional models, the individual therapist level did not account for significant variance in anxiety and depressive symptoms. We therefore chose to include only the patient level for parsimony. We first examined two-way interaction effects of age group and weeks of treatment on depression and anxiety symptoms to evaluate whether the two age groups significantly differed in their rates of change. We then examined three-way interaction effects of age group, week number, and total number of weeks on depressive and anxiety symptoms to evaluate whether the effects of engagement on the rates of change significantly differed between the two age groups. In all multilevel regression models, we controlled for baseline symptom severity (i.e., total PHQ-9 or GAD-7 scores, respectively). We also included the random intercept and random slope of session number by patients. We used the default maximum likelihood estimation method of the *nlme* package to handle missing data. We used Cohen's d to estimate effect sizes of symptom reduction from baseline to each follow-up assessment point.

As secondary measures of clinical outcome, we analyzed the following four indicators of symptom reduction at final treatment week in both cohorts: response rate (>50% reduction in PHQ-9 or GAD-7 scores), remission rate (PHQ-9 or GAD-7 scores<5), clinically significant change (>5 point reduction in scores from baseline to final treatment week), and deterioration (>5 point increase in scores from baseline to final treatment week). Sensitivity analyses examined survey non-response. All analyses were performed in R 4.1 (35) (See Tables 2, 3).

**Table 2:** Clinical outcomes for the older sample (*N* = 2,470).

**Timepoint**	**Available**	**Mean (SD)**	**Cohen's *d* (95% CI)**	**Response rate**	**Remission rate**	**Clin. Sig.** **change**	**Deteriorated**
**PHQ observations**							
Full baseline	1,945	15.65 (4.14)	–	–	–	–	–
Baseline (at Week 3)	812	15.65 (4.14)	–	–	–	–	–
Week 3	812	11.02 (5.74)	0.99 (0.91–1.08)	**269** 33.13%	**49** 6.03%	**415** 51.11%	**38** 4.68%
Week 6	460	9.95 (5.89)	1.26 (1.15–1.37)	**201** 43.70%	**36** 7.83%	**237** 51.52%	**22** 4.78%
Week 9	280	10.11 (6.11)	1.25 (1.12–1.38)	**120** 42.86%	**21** 7.50%	**154** 55.00%	**16** 5.71%
Week 12	192	9.71 (6.23)	1.36 (1.21–1.51)	**93** 48.44%	**15** 7.81%	**107** 55.73%	**13** 6.77%
Week 15	126	9.87 (6.32)	1.35 (1.16–1.53)	**57** 45.24%	**33** 26.19%	**63** 50.00%	**7** 5.56%
**GAD-7 observations**							
Full Baseline	2,054	14.73 (3.34)	–	–	–	–	–
Baseline (at Week 3)	847	14.73 (3.34)	–	–	–	–	–
Week 3	847	10.54 (5.09)	1.07 (0.98–1.15)	**259** 30.51%	**51** 6.02%	**469** 37.29%	**49** 5.79%
Week 6	449	9.42 (5.05)	1.43 (1.33–1.54)	**186** 41.43%	**36** 8.02%	**246** 48.62%	**13** 2.90%
Week 9	263	8.99 (5.10)	1.60 (1.47–1.74)	**116** 44.41%	**14** 5.32%	**136** 50.79%	**6** 2.28%
Week 12	191	8.96 (5.03)	1.64 (1.48–1.80)	**89** 46.60%	**9** 4.71%	**89** 50.00%	**5** 2.62%
Week 15	128	8.81 (5.36)	1.70 (1.51–1.88)	**61** 47.66%	**29** 22.65%	**68** 52.80%	**7** 5.47%

Rates of response, remission, clinically significant change, and deterioration are calculated using baseline scores at Week 3 compared with scores at individual timepoints.

Response rate defined as 50% or larger reduction in symptoms. Remission defined as final symptom score below 5. Clinically significant change as moving below the clinical threshold (score of <10) AND improving at least 5 points. Deterioration as worsening of symptoms by 5 or more points. Bolded values are the n, or number of participants in each cell.

**Table 3:** Clinical outcomes for the matched sample (*N* = 2,663).

**Timepoint**	**Available**	**Mean (SD)**	**Cohen's *d* (95% CI)**	**Response rate**	**Remission rate**	**Clin. sig.** **change**	**Deteriorated**
**PHQ observations**							
Full baseline	2,050	15.34 (4.22)	–	–	–	–	–
Baseline (at Week 3)	917	15.39 (4.21)	–	–	–	–	–
Week 3	917	10.84 (5.72)	0.95 (0.87–1.03)	**281** 30.64%	**66** 7.20%	**488** 53.22%	**55** 5.99%
Week 6	443	9.98 (5.88)	1.18 (1.07–1.29)	**173** 39.05%	**33** 7.45%	**254** 57.34%	**31** 6.99%
Week 9	243	9.51 (5.94)	1.32 (1.18–1.46)	**106** 43.62%	**19** 7.82%	**143** 58.85%	**17** 6.99%
Week 12	157	8.03 (4.97)	1.71 (1.54–1.88)	**81** 51.59%	**15** 9.55%	**98** 62.42%	**3** 1.91%
Week 15	103	7.64 (5.88)	1.79 (1.58–1.99)	**55** 53.40%	**36** 34.95%	**62** 60.19%	**5** 4.85%
**GAD-7 observations**							
Full Baseline	2,256	14.86 (3.31)	–	–	–	–	–
Baseline (at Week 3)	989	14.83 (3.26)	–	–	–	–	–
Week 3	989	10.50 (4.84)	1.14 (1.06–1.22)	**301** 30.43%	**42** 4.25%	**489** 49.44%	**37** 3.74%
Week 6	485	9.61 (5.03)	1.43 (1.33–1.54)	**197** 40.62%	**34** 7.01%	**255** 52.58%	**19** 3.92%
Week 9	275	8.72 (4.85)	1.75 (1.62–1.89)	**117** 42.55%	**18** 6.55%	**129** 46.91%	**10** 3.64%
Week 12	177	7.85 (4.48)	2.06 (1.90–2.23)	**97** 54.80%	**15** 8.47%	**91** 51.41%	**3** 1.69%
Week 15	115	7.66 (4.74)	2.13 (1.93–2.32)	**65** 56.52%	**26** 22.61%	**58** 50.43%	**2** 1.74%

Rates of response, remission, clinically significant change, and deterioration are calculated using baseline scores at Week 3 compared with scores at individual timepoints.

Response rate defined as 50% or larger reduction in symptoms. Remission defined as final symptom score below 5. Clinically significant change as moving below the clinical threshold (score of <10) AND improving at least 5 points. Deterioration as worsening of symptoms by 5 or more points.

## Results

The cohort of older users was matched by treatment date to an otherwise random sample of younger users. Both cohorts were 76% female. There was a statistically significant difference (*p* = 0.009) in the ethnic breakdown between the two cohorts: 85% of the older cohort vs. 65% of the younger cohort identified as Caucasian (see Table 1). There were no significant differences between cohorts on baseline symptom scale scores.

### Treatment engagement

Patients in the older age group remained on the platform for a significantly greater duration (93 days) than younger adults (81 days) (*b* = −0.56, *SE* = 0.19, *p* = 0.003) (Table 6). Older adults, however, did not actively message on the platform for significantly more days (18.93 vs. 18.43 days, respectively). Weekly messages also did not differ, with an average of 3.67 for older adults (314.5 mean words, SD = 720.2) and 4.87 for younger adults (336.17 mean words, SD = 747.1). Age as a continuous variable was not associated with any metric of treatment engagement (Pearson r's range: −0.034 to 0.006).

Among those who provided reasons for leaving therapy prior to 15 weeks (*n* = 538), the most frequently reported for both age groups were cost, meeting their goals for care, or not finding the approach helpful (Table 4). Older adults were significantly more likely than younger adults to report discontinuing due to meeting their goals for care, and less likely to discontinue due to cost.

**Table 4:** Primary reason for therapy discontinuation.

	**Older users (*****n*** = **2,310)**		**Matched sample (*****n*** = **2,663)**		**Fisher's exact test**	
	***n***	**%**	***n***	**%**	***p*-value**	**95% CI**
Cancellation reasons						
Cost	109	34.49%	116	49.79%	0.0004^***^	1.31–2.70
Lost interest	15	4.75%	1	0.43%	0.003^**^	0.0021–0.57
Move to face-to-face	6	1.90%	2	0.86%	0.48	0.044–2.53
Tech	17	5.38%	10	4.29%	0.69	0.32–1.87
Met goal	108	34.18%	60	25.75%	0.039	0.45–0.97
Privacy	1	0.32%	0	0.00%	1	0.00–52.85
Therapist unresponsive	17	5.38%	11	4.72%	0.85	0.36–2.02
Not helpful	41	12.97 %	32	13.72%	0.80	0.63–1.81
Other	17	5.38%	1	0.43%	–	–
Did not respond	1,994		2,430			

Because the data was not normally distributed according to a Shapiro-Wilk test, a Fisher's Exact Test was used over a Chi-Squared Test.

** p < 0.01, and

*** p < 0.001.

### Symptom reduction

Older and younger adults did not differ in their final depressive (*b* = 0.10, *SE* = 0.24, *p* = 0.666) or anxiety symptoms (*b* = 0.31, *SE* = 0.21, *p* = 0.138), or when controlling for total number of weeks in treatment. Patients in the younger age group experienced a greater rate of change than older adults in their anxiety (*b* = −0.07, *SE* = 0.03, *p* = 0.031), but not depressive symptoms (*b* = −0.05, *SE* = 0.04, *p* = 0.133). Patients with fewer total treatment weeks experienced smaller rates of change in both depressive (*b* = 0.07, *SE* = 0.01, *p* < 0.001) and anxiety symptoms (*b* = 0.06, *SE* = 0.01, *p* < 0.001). Rates of change in depressive symptoms among patients in the younger age group, however, were less negatively affected by their total number of weeks (*b* = −0.02, *SE* = 0.01, *p* = 0.048). Fixed main and interaction effects of the two-way and three-way interaction models for depressive and anxiety symptoms are presented in Tables 5, 6, respectively.

**Table 5:** Fixed main and interaction effects of age group and total number of weeks on rates of change in depressive symptoms.

**Variable**	**Model 1**					**Model 2**				
	***b***	***SE***	***t***	***p***	***d***	***b***	***SE***	***t***	***p***	***d***
Week number	−0.47	0.03	−18.8	< 0.001	−0.55	−1.47	0.09	−16.07	< 0.001	−0.48
Age group	0.19	0.28	0.68	0.500	0.03	0.41	0.77	0.53	0.593	0.02
Total number of weeks						−0.21	0.04	−4.82	< 0.001	−0.21
Week number × Age group	−0.05	0.04	−1.50	0.133	−0.04	0.24	0.13	1.93	0.054	0.06
Week number × Total number of weeks						0.07	0.01	11.17	< 0.001	0.33
Age group × Total number of weeks						−0.09	0.06	−1.42	0.157	−0.06
Week number × Age group × Total number of weeks						−0.02	0.01	−1.98	0.048	−0.06

The younger age group is the reference for the dichotomous variable.

**Table 6:** Fixed main and interaction effects of age group and total number of weeks on rates of change in anxiety symptoms.

**Variable**	**Model 1**					**Model 2**				
	***b***	***SE***	***t***	***p***	***d***	***b***	***SE***	***t***	***p***	***d***
Week number	−0.46	0.02	−21.42	< 0.001	−0.63	−1.38	0.08	−17.31	< 0.001	−0.51
Age group	0.77	0.23	3.36	< 0.001	0.15	0.28	0.65	0.43	0.664	0.02
Total number of weeks						−0.27	0.04	−7.51	< 0.001	−0.33
Week number × Age group	−0.07	0.03	−2.16	0.031	−0.06	0.16	0.11	1.45	0.147	0.04
Week number × Total number of weeks						0.06	0.01	11.96	< 0.001	0.35
Age group × Total number of weeks						0.00	0.05	−0.01	0.994	0.00
Week number × Age group × Total number of weeks						−0.01	0.01	−1.76	0.079	−0.05

The younger age group is the reference for the dichotomous variable.

Tables 2, 3 present rates of depression and anxiety treatment response, remission, clinically significant change, and deterioration. The response rate of older adults was 45.24% for depression and 47.66% for anxiety, and of younger adults was 53.40% for depression and 56.52% for anxiety. Mixed modeling suggested that any differences between age cohorts in these rates were not meaningful by the end of treatment.

## Discussion

The principal findings of this study were that among individuals willing to initiate care through a DMHI, older adults had overall similar engagement as younger adults and similar improvement in symptoms of depression and anxiety. Older patients attended an average of 12 more days of treatment than younger patients, but there were no differences in number of days actively messaging on the platform, number of messages per week, or word count per week. Each age group showed clinically meaningful response and remission rates for both depression and anxiety. While older adults needed three additional weeks to report final anxiety scores similar to younger adults, the two groups did not differ in reduction of depressive or anxiety symptoms over the 15-week treatment period. These reductions were not accounted for by length of time in treatment nor by any measure of treatment engagement we examined such as number of words or messages sent.

The study's findings on treatment engagement for older adults, who attended treatment for an average of 93 days (13.3 weeks), support the accessibility of such digital interventions. This attendance rate is consistent with a prior study of a large sample of predominantly younger adults using the same telemedicine platform, which found that 59% completed a predetermined endpoint of 12 weeks of treatment (28). Our finding is also consistent with the average treatment length for in person psychotherapy as represented in both psychotherapy studies and treatment manuals. Time in treatment exceeded that typically found in self-guided digital mental health applications, where a systematic review estimated the median 30-day app retention rate to be only 3.9% (37). Moreover, engagement rates in our sample exceeded those found in real world face-to-face psychotherapy. A large study of individuals with major depression seeking care in the community mental health system (38) reported a median of 5 and a mode of one psychotherapy session.

Available data on reasons for discontinuation prior to 15 weeks showed similar responses across age groups; older adults, however, were significantly more likely than younger adults to report discontinuing due to meeting their goals for care, and less likely to discontinue due to cost. While only 5.38% of older adults reported that they discontinued treatment due to difficulties with technology, lack of response to this question posed *via* the digital platform could be due to any number of reasons including having difficulty with technology. In addition, the participant pool was likely skewed toward those with more technology experience and comfort. Without considerations of support for those who don't use technology, DMHIs may exacerbate a divide among those already who tend to be the most marginalized (e.g., older adults with less support, lower income, less technology experience, etc.). Message-based care, however, does have a few advantages over app-store DMHIs, including that it is not limited to texting but can be provided *via* voicemail and email, technologies that most older adults are socialized to use. In addition, the message-based interface is Americans with Disabilities Act (ADA)-friendly. The telemedicine platform examined conforms to the Web Content Accessibility Guidelines (WCAG) with tools such as mouse and keyboard replacements, voice recognition, speech enablement, and hands-free/touch-free navigation. Thus, overall findings on treatment engagementare promising in showing that participants benefited from a DMHI designed for general use, with no specific focus given to adaptations for older adults.

The study's second primary finding was that there were no significant differences in 15-week symptom severity reduction between older and younger adults. Specifically, 45% of depressed older adults responded to treatment, in comparison to 53% of younger adults. Similarly, 48% of older adults with elevated anxiety responded, in comparison to 57% of younger adults. These clinical outcomes were consistent with a prior study of predominantly younger adults using the same telemedicine platform (28). Large effect sizes found in the current study compare favorably to the small to medium effect sizes found in meta-analyses of app-supported DMHIs compared (23), andat least comparable to or larger than the medium to large effect sizes for depression and anxiety found in traditional in-person psychotherapy studies of both younger and older adults (39–43). Future research is needed to confirm these observations on effect sizes *via* different psychotherapy modalities.

Strengths of the study include a large sample size of older and matched younger patients; representation from urban and rural settings across the United States; and ability to naturalistically examine treatment engagement and clinical outcomes among older adults, an underserved and growing population. Study limitations include large amounts of missing data to characterize important sociodemographic characteristics of the sample and on clinical outcomes at all follow up points. Missing data were due to random factors, i.e., people who chose not to fill out the surveys, and missing data not at random, i.e., people who left the platform due to improving, disliking the service, or other reasons that prevented them from continuing to use it. To account for the missing data, our statistical approach utilized likewise deletion for all analyses but the multilevel modeling. To minimize missing data in our multilevel models, our statistical approach took into account length of treatment and dropout as an interaction for outcome expectancy. Secondly, the study was not a controlled trial capable of concluding that the intervention as delivered is truly effective. Our design limits us to comparing the clinical outcome patterns of the older and younger patient cohorts. Thirdly, findings may be generalized to those individuals willing to initiate care through a DMHI for their depression or anxiety. As participants likely had greater technology experience and comfort than those who were not enrolled, future research may consider the benefits of further strategies to engage and support those who do not use such technology.

In summary, these results suggest that asynchronous messaging as an emerging DMHI performs similarly for older adults relative to younger cohorts across metrics of engagement and clinical outcomes. These findings also match other findings to date showing consistent and predictable outcomes across a variety of geographic areas and demographic groups. Given the advantages of message-based care for aiding a mental health workforce in serving larger numbers of individuals in need and the expected growth of the aging population, these findings could help healthcare systems in evaluating a variety of treatment options and delivery media for meeting the healthcare needs of the future.

## Data availability statement

Talkspace provided data for the study. The data are not publicly available due to HIPPA restrictions (e.g., information that could compromise the privacy of research participants). The raw data supporting will be made available by the authors, PR and NF, without undue reservation.

## Ethics statement

The studies involving human participants were reviewed and approved by Institutional Review Board at Teachers College, Columbia University. The patients/participants provided their written informed consent to participate in this study.

## Author contributions

PR, TH, GA, and PA: design and conduct of the study. JS, NF, PR, GA, and PA: collection, management, analysis, and interpretation of the data. PR, NF, JS, TH, GA, and PA: preparation, review, or approval of the manuscript, and decision to submit the manuscript for publication. All authors contributed to the article and approved the submitted version.

## Funding

Funding was provided by Talkspace, NIMH R44MH124334, and NIMH P50MH115837.

## Conflict of interest

Authors NF and TH are employees of the Talkspace platform. The remaining authors declare that the research was conducted in the absence of any commercial or financial relationships that could be construed as a potential conflict of interest.

## Publisher's note

All claims expressed in this article are solely those of the authors and do not necessarily represent those of their affiliated organizations, or those of the publisher, the editors and the reviewers. Any product that may be evaluated in this article, or claim that may be made by its manufacturer, is not guaranteed or endorsed by the publisher.
